# Perfluoropolyether (PFPE) Intermediate Molds for High-Resolution Thermal Nanoimprint Lithography

**DOI:** 10.3390/nano8080609

**Published:** 2018-08-10

**Authors:** Cecilia Masciullo, Agnese Sonato, Filippo Romanato, Marco Cecchini

**Affiliations:** 1 National Enterprise for nanoScience and nanoTechnology (NEST), Scuola Normale Superiore and Istituto Nanoscienze-CNR, Piazza San Silvestro 12, 56127 Pisa, Italy; cecilia.masciullo@sns.it; 2 Consiglio Nazionale delle Ricerche-Istituto Officina dei Materiali (CNR-IOM), Area Science Park, S.S. 14, km 163.5, 34149 Basovizza (TS), Italy; agnese.sonato@gmail.com (A.S.); filippo.romanato@unipd.it (F.R.)

**Keywords:** PFPE, nanoimprint, hot embossing, nanograting, intermediate mold, perfluoropolyether, soft lithography

## Abstract

Among soft lithography techniques, Thermal Nanoimprint Lithography (NIL) is a high-throughput and low-cost process that can be applied to a broad range of thermoplastic materials. By simply applying the appropriate pressure and temperature combination, it is possible to transfer a pattern from a mold surface to the chosen material. Usually, high-resolution and large-area NIL molds are difficult to fabricate and expensive. Furthermore, they are typically made of silicon or other hard materials such as nickel or quartz for preserving their functionality. Nonetheless, after a large number of imprinting cycles, they undergo degradation and become unusable. In this paper, we introduce and characterize an innovative two-step NIL process based on the use of a perfluoropolyether (PFPE) intermediate mold to replicate sub-100 nm features from a silicon mold to the final thermoplastic material. We compare PFPE elastomeric molds with molds made of the standard polydimethylsiloxane (PDMS) elastomer, which demonstrates better resolution and fidelity of the replica process. By using PFPE intermediate molds, the nanostructured masters are preserved and the throughput of the process is significantly enhanced.

## 1. Introduction

Thermal nanoimprint lithography (NIL) [1,2] is a high-throughput and low-cost soft lithography technique by which a surface pattern on a typically hard mold is physically imprinted into a thermoplastic material, which is often a polymer. The polymer can be deposited as thin film on a substrate or used as a free-standing thicker foil (in this last case, the process was originally introduced as “hot-embossing”. However, very often NIL and hot-embossing are used in the literature with the same meaning when the replicated features are of sub-micrometric dimension. In this paper, we use the term NIL to refer to both cases). The process is based on heating the material above its glass transition temperature (T_g_) and making it flow into the mold cavities by applying an adequate pressure. After cooling down below T_g_, the pressure is released to complete the replica process. 

Usually, high-resolution and large-area NIL molds are expensive and difficult to fabricate. Even though they are typically made of silicon or other hard materials like nickel or quartz, after a number of imprinting cycles, they start cracking and, therefore, become unusable. In order to preserve the mold without affecting the throughput, intermediate molds were introduced. Intermediate molds are replicas of the original mold that are themselves used as molds to transfer the topographies to the final material. This interest is also present at an industrial level. For example, the company Obducat AB has recently patented [3] an imprinting apparatus to perform a two-step process involving intermediates for typical topographies ranging from gratings of 80 nm linewidth up to micrometric pillars.

Intermediate molds are generally produced in plastic or soft materials by soft-lithography techniques and must guarantee high-fidelity copies and a sufficiently high number of processes before undergoing degradation [4]. While polymers such as poly-(methyl-methacrylate) (PMMA) or polystyrene (PS) are frequently used in thermal NIL, they are not recommended as material for intermediate molds because they are generally sticky and tend to crack during the release step [5]. Differently, beyond the ease of fabrication, elastomeric materials can ensure good elastic adaptation and conformal contact with the substrate, which leads to intimate contact without voids [6].

Polydimethylsiloxane (PDMS) is widely used as soft-mold material because of its low surface energy and mechanical properties, which allow conformal contact and easy release from the initial mold and the final imprinted film. However, the low Young’s modulus of PDMS often limits the replica process if the topographies are very small (i.e., hundreds of nm or less) and with high spatial density. Furthermore, PDMS often degrades after a few cycles of patterning [5,7]. Since their high stiffness, high temperature resistance, and unique anti-adhesive properties, fluorinated polymers have optimal properties for soft plastic molds fabrication. Barbero and co-workers [8] demonstrated that ethylene(tetrafluoroethylene) (ETFE) can sustain multiple embossing (at T = 160 °C and P ≅ 1 bar) to transfer gratings (period = 2 µm, and ridge width = 700 nm) to PMMA films. However, owing to the typical dimension of its crystallization domains (100 nm) [9], ETFE allowed a maximal resolution of ≈150 nm. In the paper of Greer coworkers [10], Teflon^®^ fluorinated ethylene–propylene (FEP) was exploited as material for soft-nanoimprint lithography. They could transfer 45 nm-diameter pits to the nano-imprint specific resist mr-NIL210 XP and to the photoresist Nano SU-8 3005, but the low FEP glass transition temperature (80 °C) greatly limited the number of compatible thermoplastic materials.

Perfluoropolyether (PFPE)-based elastomers are a unique class of fluorinated polymers whose structure is formed by linear chains based on multiple strong carbon fluorine bonds, which entail high stiffness and temperature resistance. Prior curing fluoropolymers are viscous liquids at room temperature and are characterized by a very low surface energy. This facilitates the filling of nanoscale cavities and guarantees an anti-adhesive behavior [11]. PFPEs are inert and exhibit high durability and toughness, high gas permeability, and low toxicity [12,13], with additional features of chemical and thermal stability. These characteristics minimize degradation under use and provide good lubricity, which reduces the contact surface wear. PFPE-based elastomers are, therefore, promising for NIL but only very rarely used to this end. In Reference [11], PFPE was successfully tested against silicon and PDMS as mold for thermal nanoimprinting of polycarbonate (PC) sheets. More specifically, the process was performed at T = 170 °C and P = 5 bar for transferring gratings with period of 1 μm (250 nm depth), 400 nm (330 nm depth), and 300 nm (330 nm depth). They also stated that, owing to its gas permeability, PFPE molds prevent air-trapping issues, which allowed for the use of lower imprint pressures [14]. However, a long-time process (≅30 min) at a rather high temperature (20° more than PC T_g_ = 150 °C) were required to fabricate a high-fidelity replica. To the best of our knowledge, only in a short conference proceeding PFPE was exploited to fabricate an intermediate mold, but as a film on a silicon substrate. The patterning was performed by casting and thermal-curing [15]. In this paper, the initial mold was a silicon substrate with a 200-nm-period grating (with 120-nm-depth) on its surface and the final replicas were obtained in poly-(vinyl phenyl ketone) (PVPK) by thermal NIL (T = 90 °C and P = 70 bar). The authors found that PFPE molds could successfully transfer the grating pattern and also replicate the roughness present along the ridge edges. For this reason, they speculated that PFPE could in principle be suitable for features with dimension far beyond 100 nm. However, the intermediate mold fabrication process was rather long (1–3 h of annealing) and led to very poor adhesion of the fluoropolymer to the silicon support [15]. This last issue might compromise the final NIL step.

In this paper, we propose and characterize by using a scanning electron microscopy and an atomic force microscopy through a two-step process based on the use of a free-standing patterned film of perfluoropolyether (PFPE) as an intermediate mold to replicate sub-micrometer features from a silicon mold to the final thermoplastic polymer. We compare PFPE elastomeric molds with more standard molds made of PDMS, which demonstrates better resolution and fidelity of the replica process. Lastly, we test PFPE molds for transferring isolated grooves and ridges with sub-100-nm lateral dimension.

## 2. Experimental

*Mold fabrication.* Molds with sub-100-nm resolution were obtained in PMMA by electron-beam lithography (EBL) starting from commercial p-doped silicon wafers (SYLTRONIX, Archamps, France). Each mold was initially processed by EBL to generate arrays of ridges and grooves of 100 mm^2^ area into a 50-nm-thick PMMA film. PMMA was spun over a 5 nm-thick titanium layer and was previously deposited on the silicon wafer by thermal evaporation. After cleaning with nitrogen flow, the molds were systematically characterized by optical microscopy (Carl Zeiss Microscopy, Jena, Germany) and atomic-force microscopy (Veeco Instruments Inc., Santa Barbara, CA, USA). The mold with 600-nm-period grating was fabricated by using laser interference lithography (LIL). SPR220 (Microposit, Shipley European Limited, Shipley, UK) was spun onto a silicon wafer with a spin speed of 4000 rpm for 30 s. The sample was exposed to a 50 mW helium cadmium (HeCd) laser, which emitted a TEM_00_ single mode at a 325 nm light source with a beam incidence angle of 165.7° and an exposure dose of 77 mJ/cm. Resist developing was performed by immersing the sample in an MF319/Milli-Q water (10:1) solution for 15 s [16].

*Polydimethylsiloxane (PDMS) intermediate mold fabrication*. The precursor PDMS polymer (SYLGARD 184, The Dow Chemical Company, Pittsburg, CA, USA) was mixed with its curing agent at a ratio of 10:1 and poured onto the nanostructured mold. The uncured replica was then left resting for 10 min to reduce surface inhomogeneities. Then it was baked in an oven for 2 h at 80 °C. After thermal curing, the replica was gently removed from the master using scalpel and tweezers [17].

*PerFluoroPolyEther (PFPE) intermediate mold fabrication*. PFPE resin (FLUOROLINK^®^ MD 700, Solvay Speciality Polymers, Bollate, Italy) was mixed with 3% *w*/*w* photoinitiator Darocure 1173 (C_10_H_12_O_2_, 405655 Sigma Aldrich, Milano, Italy), poured on top of the PMMA surfaces, and crosslinked by UV-light (365 nm, 25 mW·cm^−2^). The exposure was performed in two steps, which was reported in References [18,19]. Briefly, the samples were kept for 180 s in nitrogen atmosphere and then were kept for 60 s in air. After curing, the PFPE films were easily peeled off and cleaned with nitrogen flow.

*Cyclic Olefin Copolymer (COC) nanoimprinting*. COC foils (thickness 140 µm, Microfluidic ChipShop GmbH, Jena, Germany) were imprinted using an Obducat Nanoimprint 24 system (Obducat, Lund, Sweden) with the PFPE intermediate molds. After cleaning with 2-propanol, the COC substrates were placed on top of the molds and softened by raising the temperature up to 150 °C. A pressure of 50 bar was then applied for 300 s before cooling down to 70 °C, i.e., below the glass transition temperature of the copolymer (T_g_ = 134 °C). Lastly, the pressure was released and the mold was detached from the imprinted COC with a scalpel [20].

*Scanning Electron Microscopy*. Molds, intermediate molds, and final replicas were analyzed with a LEO 1525 field emission scanning electron microscope. In order to enhance the topography of substrate surfaces, image acquisition was carried out by using an Everheart-Thornley detector.

*Contact Angle Measurements.* Substrate wettability was evaluated by contact angle measurements acquired with a CAM 200 instrument (KSV Instruments, Helsinki, Finland). A deionized water drop was deposited on top of each substrate through a micro-syringe. All these measurements were performed in air at room temperature. Data are reported as mean ± SD.

*Atomic Force Microscopy*. Sample topographies were characterized by an atomic force microscope (Veeco Innova Scanning Probe Microscope, Veeco Instruments Inc., Santa Barbara, CA, USA), operating in tapping mode. The scan frequency was set at 0.977 Hz and the scanning areas were 5 × 5 μm^2^_._ At least three areas were analyzed per sample (512 point/line each). At least three PFPE intermediate mold and COC replicas were imaged for each topography type. A silicon nitride tip with a nominal spring constant in the range of 0.2–0.8 N/m and a resonant frequency of 45–95 kHz was used. All the measurements were performed in air at room temperature and raw scan data were leveled by surface subtraction to remove possible sample tilts. Data were analyzed by the Gwiddion software (Gwiddion 2.47 version, Brno, Czech Republic, “Profile” tool) and reported as mean ± SD. Full Width Half Maximum values were measured from the AFM profiles by the “Analysis: Peaks and Baseline: Multiple Peak Fit- Gaussian fit” tool of the software Origin (OriginLab Corporation, Northampton, MA, USA https://www.originlab.com, version 9.0).

*Statistical analysis.* Data are reported as average values ± the standard deviation (mean ± SD). Data were statistically analyzed by the GraphPad PRISM 6.1 program (GraphPad Software, San Diego, CA, USA). Student’ *t*-test (unpaired) analysis was used to compare distributions. The mean values obtained in each repeated experiment were assumed to be normally distributed regarding the true mean. Statistical significance refers to results where *p* < 0.05.

## 3. Results and Discussion

In this study, we have employed perfluoropolyether (PFPE) as material for intermediate molds for thermal nanoimprinting and compared it with the more common polydimethylsiloxane (PDMS). We started from nanostructures of polymethyl-methacrylate (PMMA) or Microposit SPR220 photoresist/PGMEA (1:2) with the final goal to obtain copies in Cyclic Olefin Copolymer (COC) and determine the minimal feature size that could be replicated by this two-step process with high-fidelity.

### 3.1. Step 1: PFPE Intermediate Mold Fabrication and Characterization

**Figure 1:**
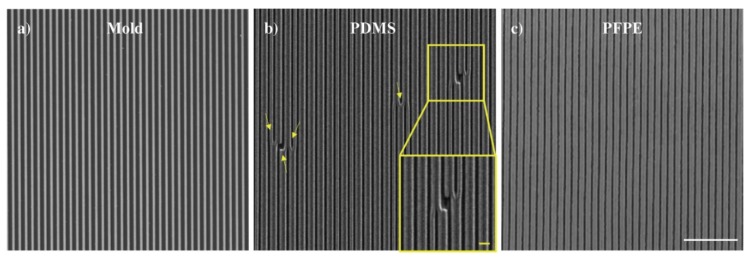
Scanning electron microscope representative images of (**a**) the 600 nm-periodic nano-grating initial mold, (**b**) the PDMS replica, and (**c**) the PFPE replica. Scale bar: 5 μm. Yellow arrows highlight the presence of defects in the PDMS replica. Inset of (**b**) zoomed image of a representative area with defects, scale bar = 1 μm.

First, after having fabricated a polymeric nanograting as an initial mold (ridge width = groove width = groove depth = 300 nm, Figure 1a) by laser interference lithography (LIL), we applied replica-molding protocols (see Materials and Methods for details) to obtain copies of the initial mold in two elastomeric materials: PFPE and the standard PDMS. Usually, PDMS is used as an elastic stamp to transfer micro-patterns or nano-patterns using NIL or soft lithography techniques [2], but it is also commonly used to fabricate micro-scale fluidic systems such as separation systems, micro mixers, or micro channels [17,21]. Features of several hundreds of nm in close proximity and with high spatial density are challenging to replicate over large areas in elastomeric materials and, for this reason, this kind of pattern was chosen to compare PFPE and PDMS. Figure 1a–c report representative SEM images of the initial mold and of PDMS and PFPE replicas, respectively. As highlighted in Figure 1b, the nanograting could not be successfully reproduced in PDMS. More specifically, we noticed a widespread collapse of subsequent ridges and the presence of many hole-cracks along the grating lines (*yellow arrows and inset* in Figure 1b). Failure in replicating patterns with PDMS can be caused by its rather low Young’s modulus (≈1.5 MPa) [22], which can lead to lateral collapse by merging and buckling dense imprinted structures when the features have lateral dimensions that are generally smaller than ≈ 500 nm and have a high aspect ratio (greater than ≈1) [5,6]. These issues can be avoided with PFPE, which has a higher Young’s modulus (≈10 MPa) [23]. As depicted in Figure 1c, the PFPE replica shows nanograting profiles over the whole cm^2^ area without the imperfections or damages that were found in those made of PDMS.

Typically, two forces exerted during the detachment of the replica from the mold must also be minimized to enhance replication fidelity and extend mold lifetime: friction forces applied to the pattern side-walls and adhesion forces between the two surfaces [24]. Having PFPEs lower surface energy than PDMS (typical values are ≈12.7 mJ/m^2^ [23] for PFPE and ≈25 mJ/m^2^ for PDMS) [25], these materials can reduce the mentioned friction and adhesion forces, which proves overall better performance with respect to PDMS. Solely because of this characteristic and of their inertness, we should also mention that PFPEs are excellent lubricants for reactive and aggressive environments. Typical applications are greased for aircraft instrument bearing and for semiconductor processing or plasma etching equipment lubricant. In order to better characterize our intermediate molds, we evaluated their wettability by performing water contact angle measurements. The contact angle was 93 ± 5°, which was in line with what was reported by other authors for PFPE substrates [26].

**Figure 2:**
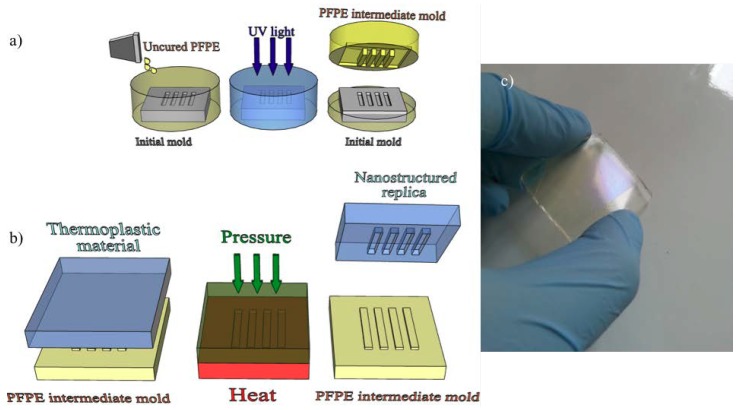
Schematic illustration of (**a**) PFPE intermediate mold fabrication and (**b**) subsequent thermal NIL process made by using the PFPE intermediate mold. The thermoplastic material used in this study is COC. (**c**) Photo of a PFPE intermediate mold.

Given the results presented in this study and due to its rigidity and low surface energy, we have selected PFPE as the material of choice for intermediate molds for thermal NIL. Our fabrication protocol (see Materials and Methods for details and Figure 2) provided PFPE elastomeric replicas of ≈3 mm thickness with a Young’s modulus of about 10 MPa [23]. This value made it compatible with standard imprinting protocols (pressures of tens of bars) and did not lead to cracking issues during mold-replica detachment. If necessary, the final thickness of PFPE can be modified by varying the volume of the mixture poured on top of the mold.

### 3.2. Step 2: COC Thermal Nanoimprinting via PFPE Intermediate Molds

In order to finally transfer the pattern to COC foils, thermal NIL was performed using PFPE intermediate molds instead of the initial ones (Figure 2b). As already mentioned, the use of an intermediate mold can significantly enhance the throughput of the replica process without damaging the initial mold. In order to characterize the fidelity of the complete two-step transfer process, we used a pattern specifically designed to test the process with extreme geometries.

We produced two new molds, which are both composed of arrays from isolated ridges and grooves defined by EBL on a PMMA film deposited on a silicon substrate. The first mold, which is named **Mold100**, has lines with nominal width of 100 nm. By the fabrication of the second mold, named **Mold50**, with a nominal linewidth of 50 nm, we wanted to test the process with sub-100-nm features. In both cases, the lines were 1-mm-long, separated by 1 µm, and the line aspect ratio (height/depth over width) was 2 and 1 for **Mold100** and **Mold50**, respectively. Isolated grooves and ridges were chosen to test the process on topographies that can represent basic building blocks for more complex layouts.

After having fabricated the PFPE intermediate molds as previously described, we performed thermal NIL to transfer the nanostructures on Cyclic Olefin Copolymer (COC) foils. The process parameters were previously optimized for this copolymer as follows: T_imprint_ = 150 °C (T^COC^_g_ = 134 °C, t = 300 s, P = 50 bar, T_cool-down_ = 80 °C) [18,19,20]. The characteristic of low surface energy of PFPE also helped in this case, thus facilitating the final detachment of the imprinted plastics. We quality checked the process by Atomic Force Microscopy (AFM) and by acquiring 3D images of the initial mold, PFPE intermediate mold, and the COC replica. Starting from 5 × 5 μm^2^ AFM images, linear profiles were extracted across ridges and grooves to measure their lateral dimension. In particular, the Full Width Half Maximum (FWHM) was used as a parameter to quantitatively compare the original nanostructures present on the mold and the ones on the PFPE intermediate and, more importantly, the ones on the COC final replica.

**Figure 3:**
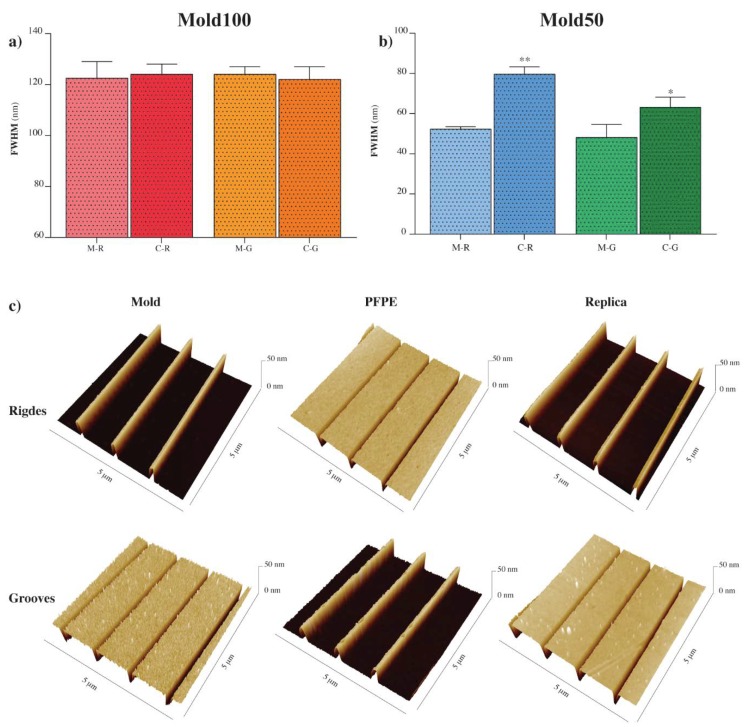
Atomic force microscopy measurements Mold100, Mold50, PFPE intermediate molds and COC final replicas. (**a**) FWHM of Mold100 ridges (M-R) and grooves (M-G), and respective COC replica ridges (C-R) and grooves (C-G). (**b**) FWHM of Mold50 ridges (M-R) and grooves (M-G) and respective COC replica ridges (C-R) and grooves (C-G). Data in (**a**,**b**) are mean ± SD, */** *p* < 0.05/0.01, unpaired *t*-test. (**c**) Representative 3D AFM images for ridges and grooves of the Mold100, PFPE intermediate mold, and COC final replica.

In case of **Mold100**, mold ridges (M-R) and mold grooves (M-G) had FWHM values of 122 ± 4 nm and 124 ± 2 nm, respectively. COC Ridges (C-R) and Grooves (C-G) showed equivalent width values (124 ± 2 nm for C-R, 122 ± 3 nm for C-G) (Figure 3a), which demonstrates that the process can successfully transfer isolated features with a lateral dimension of the order of 100 nm and aspect ratio = 2.

3D AFM reconstructions in Figure 3c show a larger view of the topographies, which confirms the satisfactory compliance of each transfer step to the previous and highlighting the consistent profiles of the original mold and COC replica. Owing to the possibility to fabricate more copies of the same initial mold and to the fact that a single PFPE intermediate mold can sustain tents of thermal imprint cycles without cracking or affecting the final feature resolution, the entire process yield is considerably increased.

**Mold50** was then tested in order to look for the process resolution limit. In this case, we measured a FWHM of 52 ± 1 nm for M-R and 48 ± 4 nm for M-G, while an enlargement was measured for the COC replica. More specifically, the FWHM was 80 ± 2 nm for C-R and 63 ± 3 nm for C-G (Figure 3c). Sub-100 nm topographies were, therefore, transferred to COC. This experiment also suggests that lines of lateral dimension of ≈80 nm (for ridges) and ≈60 nm (for grooves) and with an aspect ratio = 1 can be considered as the minimum feature size allowed by our two-step replica process.

In order to improve the resolution limit, several strategies can be adopted. For example, given that replication fidelity strongly depends on the mold rigidity, the PFPE mixture could be tailored to further increase its Young’s modulus. This would be possible by simply changing the molecular weight of the macromonomer, adding functional groups, or varying the ratio between PFPE and the cross-linker molecules [11]. There are few examples in literature about modified perfluoropolyethers and their use for replicating nanostructures. Methacryloxy-functionalized PFPE (PFPE-DMA, 10 MPa of elastic modulus) could replicate lines of 140 nm width with a depth of approximately 50 nm separated by 70 nm [27]. However, also in this case, PFPE lines underwent some slight relaxation upon the release from the patterned silicon master. In a different study, silicon nanogratings could be replicated in PFPE tetramethacrylate (2K-PFPE-TMA), which is a modified PFPE-TMA with a much higher Young’s modulus (above 155 MPa). Gratings with a 120 nm period, 50% duty cycle, and 200 nm depth (aspect ratio 3.3) were replicated even though this high aspect ratio led to ridge instability and collapse [11]. Lastly, we should mention the paper of Williams [11] where the authors demonstrated PFPE sub-50 nm resolution for the replication of nano-cones. Therefore, we do believe that, overall, sub-50 nm resolution and more would be possible, but more investigations with different topographies (such as nanoposts or nanoholes), aspect ratios, spatial densities, and polymer mixes are required to allow the unravelling of the real potential of PFPEs as mold or intermediate mold material for thermal NIL.

## 4. Conclusions

In conclusion, we have introduced and characterized an innovative two-step thermal NIL process based on the use of intermediate molds made of PFPE to replicate sub-100 nm features from a silicon mold to a final thermoplastic material (COC).

PFPE elastomeric molds were compared with molds made of the more standard PDMS, which demonstrated better resolution and fidelity of the replica process. More specifically, we showed that, in case of 600-nm-period nanogratings, PDMS could not successfully reproduce the topography likely because of its rather low Young’s modulus. On the contrary, the more rigid PFPE allowed the nanograting to be successfully copied.

Lastly, in order to characterize the fidelity of the complete two-step transfer protocol, we used sub-100-nm patterns specifically designed to test the process with extreme geometries. We found that isolated lines of lateral dimension of ≈80 nm (in case of ridges) and ≈60 nm (in case of grooves) and with aspect ratio = 1 can be considered at the moment as the minimum feature size that can be transferred to the thermoplastics.

Given our results and the possibility to increase the rigidity of PFPEs by adding chemical groups or tailoring the PFPE/crosslinker mix, we believe that sub-50 nm resolution and more would be possible. However, more investigations with different topographies such as nanoposts, nanoholes, aspect ratios, and spatial densities are required to fully unravel the real potential of PFPEs as mold or intermediate mold material for thermal NIL.
